# Reversible Regulation of Polyubiquitin Gene *UBC* via Modified Inducible CRISPR/Cas9 System

**DOI:** 10.3390/ijms20133168

**Published:** 2019-06-28

**Authors:** Seung-Woo Han, Byung-Kwon Jung, So-Hyun Park, Kwon-Yul Ryu

**Affiliations:** Department of Life Science, University of Seoul, Seoul 02504, Korea

**Keywords:** ubiquitin, polyubiquitin gene, dCas9, upregulation, stress

## Abstract

Ubiquitin is required under both normal and stress conditions. Under stress conditions, upregulation of the polyubiquitin gene *UBC* is essential to meet the requirement of increased ubiquitin levels to confer stress resistance. However, *UBC* upregulation is usually observed only under stress conditions and not under normal conditions. Therefore, it has not been possible to upregulate *UBC* under normal conditions to study the effect of excess ubiquitin on cellular machinery. Recently, the CRISPR/Cas9 system has been widely used in biological research as a useful tool to study gene disruption effects. In this study, using an inducible CRISPR/Cas9 variant, a dCas9–VP64 fusion protein, combined with a single guide RNA (sgRNA) containing MS2 aptamer loops and MS2-p65-HSF1, we developed a system to increase the ubiquitin pool via upregulation of *UBC*. Although it is challenging to upregulate the expression of a gene that is already expressed at high levels, the significance of our system is that *UBC* upregulation can be induced in an efficient, reversible manner that is compatible with cellular processes, even under normal conditions. This system can be used to study ubiquitin pool dynamics and it will be a useful tool in identifying the role of ubiquitin under normal and stress conditions.

## 1. Introduction

Ubiquitin (Ub) is a highly conserved eukaryotic protein that plays diverse roles within cells [1]. The most important role of Ub is to polyubiquitinate its substrates and target them to the proteasome for degradation [2]. Degradation of these substrates occurs under both normal and stress conditions. A well-known antioxidant response pathway protein, Nrf2 (nuclear factor erythroid 2-related factor 2), is polyubiquitinated and highly prone to degradation under normal conditions, but is stabilized under oxidative stress conditions and translocates into the nucleus to serve as a transcription factor to increase the expression of antioxidant response genes [3,4,5]. However, misfolded proteins generated under oxidative stress conditions are polyubiquitinated and subsequently, targeted to the proteasome [6,7]. Therefore, Ub plays its role under diverse (normal and stress) conditions to maintain cellular protein homeostasis and its target substrates may differ depending on cellular status.

Under proteotoxic or oxidative stress conditions, many proteins tend to be misfolded. These misfolded proteins need to be cleared by the proteasome in a timely manner, otherwise their intermediates accumulate inside cells and oligomerize into non-native protein aggregates [7]. These protein aggregates are usually toxic to cells [8,9]. Therefore, under stress conditions, cellular demand for Ub is increased, as there are more proteins that need to be degraded. A rapid increase in the Ub pool is mainly achieved by the upregulation of the polyubiquitin genes, *UBB* and/or *UBC*, and does not involve the constitutively expressed Ub-ribosomal fusion genes, *UBA52* and *RPS27A*. In humans, *UBB* encodes three tandem repeats of Ub and *UBC* encodes nine tandem repeats of Ub [10,11]. These linear polyubiquitin proteins are known to be processed to monomeric moieties by a Ub-specific protease immediately after their synthesis [12,13]. Among these two polyubiquitin genes, upregulation of *UBC* is three times more efficient than that of *UBB*, assuming similar transcriptional activities. In fact, we have shown that the transcriptional activities of *UBB* and *UBC* are markedly different among different tissues and cell types [14,15,16]. Therefore, the contributions of *UBB* and *UBC* towards the total Ub pool may also be different. Even in the same tissues or cell types, their contributions are generally greater under stress conditions than those under normal conditions.

It is important that the Ub pool is maintained above certain threshold levels and these levels are dynamic depending on the status of the cell. We have previously shown that reduced levels of cellular Ub, via knockout or knockdown of the polyubiquitin gene *Ubc* in mouse cell lines, resulted in increased susceptibility to oxidative stress [7,17,18]. Intriguingly, a reduced Ub pool under normal conditions is also associated with developmental defects in the mouse brain and dysregulation of neural stem cell differentiation, with the promotion of gliogenesis and the suppression of neurogenesis [19,20]. These results suggest that Ub plays a pivotal role under both normal and stress conditions.

Although polyubiquitin genes are upregulated under stress conditions, the potential outcomes of polyubiquitin gene upregulation under normal conditions remain unclear. As the cellular Ub pool is tightly regulated, with low and high threshold levels under normal and stress conditions, respectively, it is possible that Ub levels above the threshold may not be harmful to cells, but may not be beneficial either. Alternatively, Ub levels above the threshold, under normal conditions, may play a protective role when cells encounter stress conditions, as they enable cells to more easily reach the higher levels of Ub required under these conditions.

In recent years, Cas9, an RNA-guided bacterial endonuclease, has been widely used for the engineering or editing of mammalian genomes [21,22]. When the two endonuclease activities (RuvC and HNH) of Cas9 are disabled by mutations, it can bind to its target sequence, but double-stranded DNA breaks cannot occur [23]. This inactive Cas9 is known as dCas9. Effector domains can also be fused to dCas9 so that they can be recruited to the region where dCas9 binds. Various effector domains have been used, including VP64 as an activator, KRAB as a repressor, and GFP fluorescence tags to detect the gene of interest [24]. For example, when the dCas9–VP64 fusion protein is targeted to the promoter region, it can be used to upregulate the target gene, if RNA polymerase II can interact with VP64 directly or even indirectly via mediator proteins.

In the present study, we were able to upregulate the polyubiquitin gene *UBC*, even under normal conditions, using an inducible dCas9–VP64 fusion protein capable of temporally upregulating target genes. We showed that this upregulation was completely reversible. This is of particular interest as upregulation of *UBC* has typically been observed under stress conditions, but not under normal conditions. Thus, the effect of excessive Ub under normal conditions has not yet been studied. Although ectopic expression of Ub can also increase cellular Ub levels, upregulation of the endogenous polyubiquitin gene provides an alternative approach that is more direct and more compatible with normal cellular processes. Therefore, our study using a modified inducible CRISPR/Cas9 system will provide new insights into the precise role of Ub under normal physiological conditions, as well as under stress conditions.

## 2. Results

### 2.1. Validation of Inducible dCas9–VP64 System

**Figure 1:**
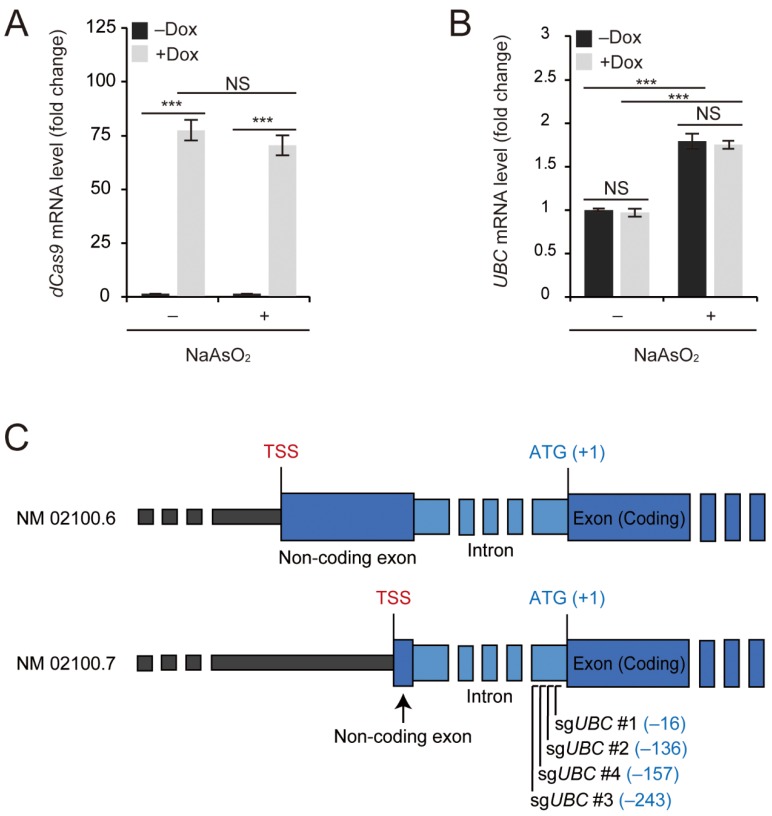
Inducible dCas9–VP64 (idCas9–VP64) system for the upregulation of the polyubiquitin gene *UBC* and the *UBC* promoter region. (**A**,**B**) Human embryonic kidney 293T (HEK293T) cells were transiently transfected with idCas9–VP64 and then treated with 5 μg/mL doxycycline (Dox) for 1 day. Before harvest, cells were treated with 10 μM NaAsO_2_ for 5 h. *dCas9* and *UBC* mRNA levels were determined by qRT-PCR (*n* = 3 each), normalized to *GAPDH*, and expressed as the fold change relative to the control (-Dox, -NaAsO_2_). (**C**) Structure of the polyubiquitin gene *UBC* based on the NCBI gene reference and indication of the designed sgRNAs targeting the intron of *UBC*. All data are presented as means ± SEM from the indicated number of samples. *** *p* < 0.001 between two groups as indicated by horizontal bars. NS: Not significant.

To validate our inducible dCas9-mediated transcriptional activation system, we first determined the levels of *dCas9* mRNA after 5 μg/mL doxycycline (Dox) treatment for 1 day. Induction with Dox resulted in a dramatic (more than 50-fold) increase in dCas9–VP64 expression in human embryonic kidney (HEK) 293T cells (Figure 1A). Increased dCas9–VP64 expression was independent of cellular stress conditions, as oxidative stress, induced by 10 μM arsenite (NaAsO_2_) treatment for 5 h, or proteotoxic stress, induced by proteasome inhibition, did not affect the induction of dCas9–VP64 (Figure 1A and Supplementary Figure S1A).

In our previous study, we showed that *UBC* is upregulated under oxidative stress induced by arsenite (13). To investigate whether Dox treatment and/or the presence of dCas9–VP64 per se influences the regulation of *UBC*, we determined their expression levels upon exposure to arsenite. Basal *UBC* expression levels were not affected by 5 μg/mL Dox treatment for 1 day and upregulation of *UBC* upon exposure to 10 μM arsenite for 5 h was independent of Dox treatment or the presence of dCas9–VP64 in the absence of sgRNAs (Figure 1B). Similarly, the presence of dCas9–VP64 in the absence of sgRNAs did not affect the upregulation of *UBC* upon proteasome inhibition (Supplementary Figure S1B). Therefore, we confirmed that inducible dCas9–VP64 (idCas9–VP64) can potentially be used to upregulate *UBC*, if the appropriate sgRNAs targeted to the regulatory region of *UBC* are identified.

### 2.2. Design of sgRNAs That can Upregulate UBC

In order to design sgRNAs that can specifically bind the regulatory region of *UBC* and recruit dCas9–VP64 for transcriptional activation, we first analyzed the promoter and coding region of *UBC* (Figure 1C). Although its expression levels differ among different cell types or tissues, *UBC* is an abundant transcript, even under normal conditions, and therefore, it may be challenging to design sgRNAs that can upregulate *UBC*. Human *UBC* is localized to chromosome 12, with the gene ID 7316 [25]. Based on the NCBI gene database, the *UBC* coding region is located in a single 2130 bp exon without any intervening sequences or introns. However, based on the NCBI Reference Sequence NM_021009.6 [26] and the recently updated NM_021009.7 [27], there is either a 455 bp or a 64 bp non-coding exon located 812 bp upstream of the coding exon (Figure 1C). Therefore, this 812 bp region, which starts with the nucleotide sequence GT and ends with AG, has long been thought to serve as an intron (14).

However, the sequences derived from these non-coding exon and intron regions have sometimes been included as part of the *UBC* promoter, used to drive the constitutive expression of structural genes, such as *dCas9* [28]. Therefore, we were curious whether this intron region plays a regulatory role in the transcriptional activation of *UBC*. To determine whether targeting a transcriptional activator to this intron region is sufficient to promote transcription by RNA polymerase II, we designed four different sgRNAs targeting the intron, between the non-coding and coding exons (Figure 1C).

**Figure 2:**
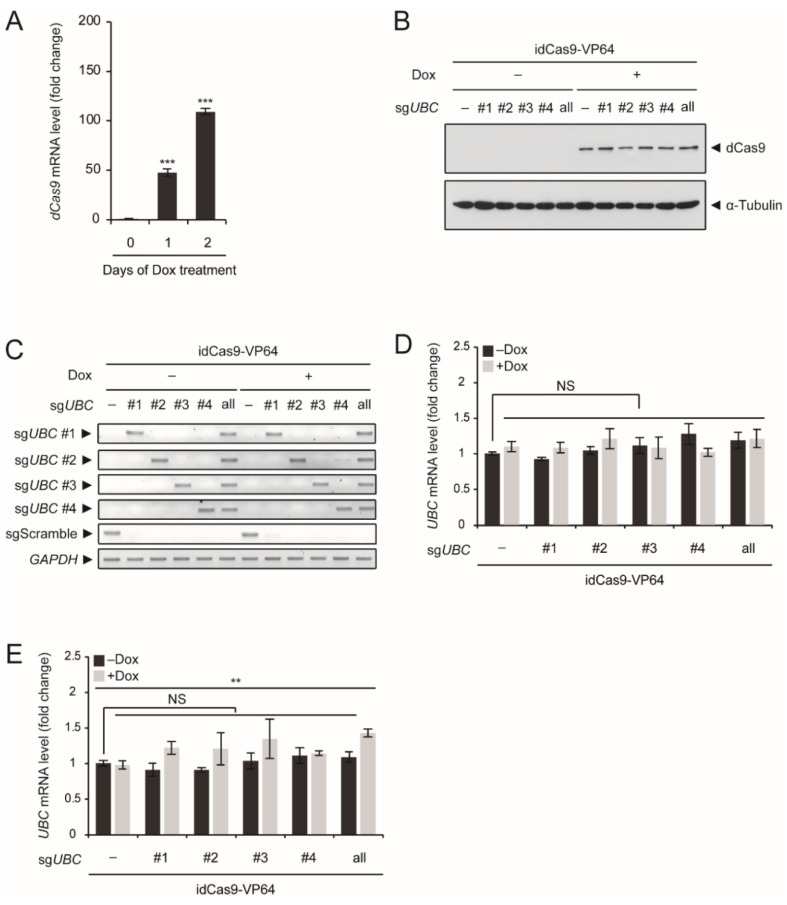
Evaluation of *UBC* upregulation using the idCas9–VP64 system with sgRNAs targeting the *UBC* intron. (**A**) HEK293T cells were transiently transfected with idCas9–VP64 and scrambled sgRNA and then treated with 5 μg/mL Dox for up to 2 days. *dCas9* mRNA levels were determined by qRT-PCR (*n* = 3 each), normalized to *GAPDH*, and expressed as the fold change relative to the control (0 days of Dox treatment). (**B**) HEK293T cells were transiently transfected with idCas9–VP64, and scrambed sgRNA (indicated as -) or sg*UBC* #1~#4, alone or in combination, and then treated with 5 μg/mL Dox for 2 days. dCas9–VP64 was detected by immunoblot analysis using an anti-Cas9 antibody. α-Tubulin was used as a loading control. (**C**) Expression of scrambled sgRNA (sgScramble) or sg*UBC* #1~#4 was determined by conventional RT-PCR. *GAPDH* was used as an internal control. (**D**,**E**) Transient transfection of HEK293T cells and Dox treatment were performed as described in (**B**). In (**E**), HEK293T cells were treated with 10 μg/mL Dox for 2 days. *UBC* mRNA levels were determined by qRT-PCR (*n* = 3 each), normalized to *GAPDH*, and expressed as the fold change relative to the control (-Dox, scrambled sgRNA). Representative immunoblots and RT-PCR results are shown from three independent experiments (**B**,**C**). Data are presented as means ± SEM from the indicated number of samples (**A**,**D**,**E**). ** *p* < 0.01; *** *p* < 0.001 vs. control (0 days of Dox treatment) or between two groups as indicated by horizontal bars. NS: Not significant.

In our system, dCas9–VP64 was inducible upon treatment with Dox. Although our long-term goal is to establish stable cell lines with sgRNA and idCas9–VP64 expression, in this study we first tested our system using transient expression of sgRNA and idCas9–VP64. After transient transfection of an idCas9–VP64 construct in HEK293T cells, higher *dCas9* mRNA levels were observed after treatment with 5 μg/mL Dox for 2 days compared to those treated for 1 day (Figure 2A). After Dox induction for 2 days, we also confirmed dCas9–VP64 protein expression via immunoblot analysis using an anti-Cas9 antibody and expression of the four different sgRNAs via RT-PCR analysis (Figure 2B,C). However, we did not observe *UBC* upregulation after transient expression of any of the tested sgRNAs, either alone or in combination (Figure 2D). Increasing the concentration of Dox from 5 μg/mL to 10 μg/mL slightly improved the results, especially when sgRNAs were expressed in combination (Figure 2E).

### 2.3. Utilization of an RNA Aptamer for UBC Upregulation

**Figure 3:**
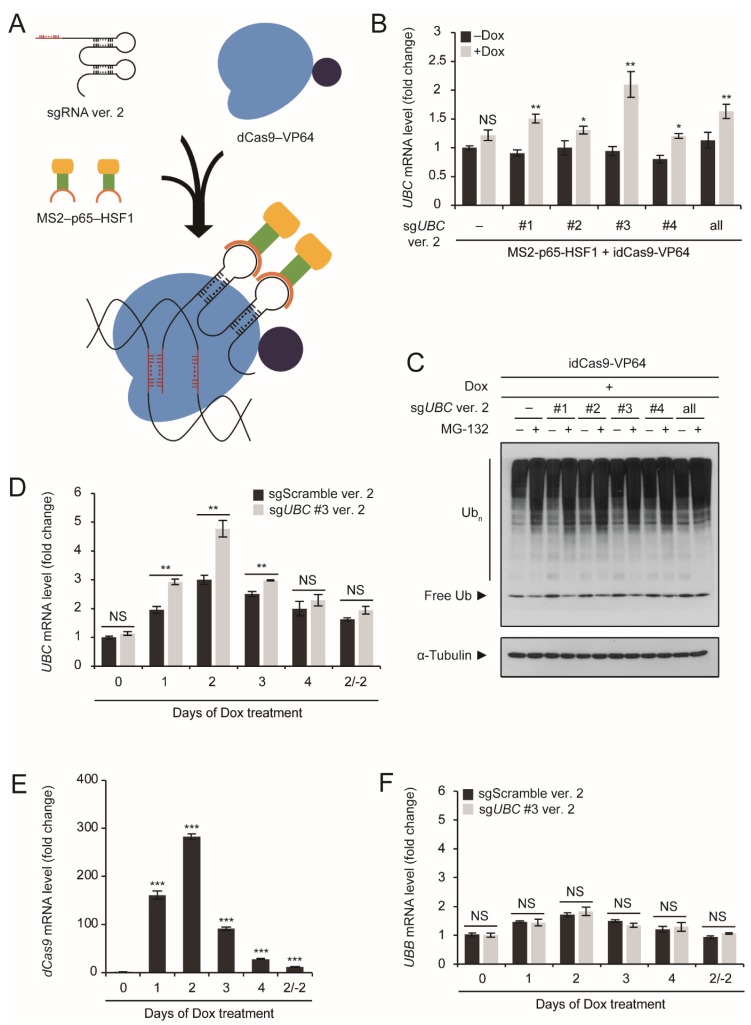
Application of an MS2 RNA aptamer and additional activators for *UBC* upregulation, based on the idCas9–VP64 system. (**A**) Schematic diagram of the working model of the MS2 aptamer and additional activators for the CRISPR activation system. MS2 protein conjugated to C-terminal activator domains, composed of the mouse NF-κB subunit (p65) and human heat shock factor 1 (HSF1), binds to the MS2 RNA stem-loop structure of sgRNA ver. 2. These sgRNA ver. 2 and MS2-p65-HSF1 complexes cooperate with idCas9–VP64 for the upregulation of target genes. (**B**) HEK293T cells were transiently transfected with idCas9–VP64, MS2-p65-HSF1, and scrambled sgRNA ver. 2 (indicated as -) or sg*UBC* #1~#4 ver. 2 (alone or in combination), and then treated with 10 μg/mL Dox for 2 days. *UBC* mRNA levels were determined by qRT-PCR (*n* = 3 each), normalized to *GAPDH*, and expressed as the fold change relative to the control (-Dox, scrambled sgRNA ver. 2). (**C**) Transient transfection of HEK293T cells and Dox treatment were performed as described in (**B**). Ub conjugates (Ub_n_) and free Ub were detected by immunoblot analysis using an anti-Ub antibody. α-Tubulin was used as a loading control. (**D**,**F**) HEK293T cells were transiently transfected with idCas9–VP64, MS2-p65-HSF1, and scrambled sgRNA (sgScramble) ver. 2 or sg*UBC* #3 ver. 2, and then treated with 10 μg/mL Dox for up to 4 days. To washout Dox, cells were treated with 10 μg/mL Dox for 2 days and it was then removed from the media for 2 days (2/-2). *UBC* and *UBB* mRNA levels were determined by qRT-PCR (*n* = 3 each), normalized to *GAPDH*, and expressed as the fold change relative to the control (0 days of Dox treatment, scrambled sgRNA ver. 2). (**E**) HEK293T cells were transiently transfected with idCas9–VP64, MS2-p65-HSF1, and sg*UBC* #3 ver. 2, and then treated with 10 μg/mL Dox for up to 4 days. Dox washout (2/-2) was performed as described above. *dCas9* mRNA levels were determined by qRT-PCR (*n* = 3 each), normalized to *GAPDH*, and expressed as the fold change relative to the control (0 days of Dox treatment, scrambled sgRNA ver. 2). Representative immunoblots are shown from three independent experiments (**C**). All data are presented as means ± SEM from the indicated number of samples. * *p* < 0.05; ** *p* < 0.01; *** *p* < 0.001 vs. control (0 days of Dox treatment or -Dox) or between two groups as indicated by horizontal bars. NS: Not significant.

To improve our system, we used sgRNAs modified with an MS2 RNA aptamer (sgRNA ver. 2) and co-expressed them with MS2-p65-HSF1, a transcriptional activator that recognizes MS2-binding loops and is recruited in close proximity to RNA polymerase II (Figure 3A). In this improved system, HEK293T cells were co-transfected with idCas9–VP64, MS2-p65-HSF1, and sgRNAs with the MS2 aptamer. Induction with 10 μg/mL Dox for 2 days resulted in a significant upregulation of *UBC* with every tested sgRNA and with sgRNA combinations (Figure 3B). Among the sgRNAs tested, sg*UBC* #3 showed the most significant upregulation of *UBC*, with more than a 2-fold increase in expression levels (Figure 3B). This upregulation resulted in increased levels of free Ub, which readily converted to Ub conjugates upon proteotoxic stress induced by proteasome inhibition (Figure 3C). Although *UBC* is generally considered a housekeeping gene, with relaxed chromatin structure (open conformation), and a transcriptionally active gene, we were able to further upregulate *UBC* using the MS2 aptamer and three transcriptional activators (VP64, p65, and HSF1). The significance of this upregulation is that it is completely artificial and is not caused by cellular stress conditions, such as oxidative stress.

Next, we tested whether the effect of sg*UBC* #3 was specific and was indeed caused by binding to its target sequence. Interestingly, when HEK293T cells were treated with 10 μg/mL Dox for up to 4 days post-transfection, *UBC* expression increased gradually during the first 2 days and then decreased thereafter, although its expression did not return to basal levels (Figure 3D). This pattern of *UBC* regulation was independent of transcriptional activation mediated by sg*UBC* #3, as it was also observed with scrambled sgRNA (Figure 3D). This correlates well with the expression patterns of dCas9, sg*UBC* #3, and MS2-p65-HSF1, which peaked at 2 days and decreased thereafter (Figure 3E and Supplementary Figure S2A,B). When cells divide, plasmid DNA constructs within cells are also distributed to their daughter cells. Therefore, repetitive cell division should result in the decreased number of transfected plasmid DNA constructs per cell. We propose that on and after 3 days post-transfection, the number of plasmid DNA constructs may be the limiting factor for transient expression of dCas9 and other components. Therefore, the reason that *UBC* upregulation does not continue more than 3 days seems to be due to the characteristics of dividing cells after transient transfection.

*UBB* expression levels were not affected by the type of sgRNA expressed (sg*UBC* #3 or scrambled sgRNA), but correlated well with dCas9 expression levels (Figure 3E,F). However, during the first 3 days post-transfection, *UBC* expression levels were significantly different depending on the type of sgRNA expressed (Figure 3D). The most dramatic upregulation of *UBC* was observed at 2 days post-transfection, when sg*UBC* #3 was expressed (Figure 3D). At 2 days post-transfection, *UBC* mRNA levels, but not *UBB* mRNA levels, increased further with the expression of sg*UBC* #3, compared to the expression of scrambled sgRNA (Figure 3D,F). Therefore, the effect of sg*UBC* #3 was specific to *UBC* expression, not to *UBB* expression, which suggests that sg*UBC* #3 exerted its effect by binding to its target sequence located in the intron of the *UBC* gene.

When Dox was washed out for 2 days after induction for 2 days, we observed a slight, but not significant, reduction in *UBC* expression levels compared to 4 days of continuous Dox treatment, with similar patterns in dCas9 expression levels (Figure 3D,E). This indicates that washing out Dox plays a role in downregulating dCas9 expression, resulting in the downregulation of *UBC*. However, prolonged culture of HEK293T cells may result in the proliferation of cells that do not harbor the idCas9–VP64 construct, which may be the major reason that dCas9 expression cannot be maintained, even with Dox induction. Nonetheless, our results suggest that the upregulation of *UBC* using sgRNA is an acute response and is completely reversible.

## 3. Discussion

In the present study, we developed a system to upregulate the polyubiquitin gene *UBC*, using an inducible CRISPR/Cas9 variant. This system is of particular interest as (1) it can upregulate the stress-responsive gene even under normal conditions; (2) it can upregulate a gene whose expression levels are already high; and (3) its upregulation is dependent on the levels of Cas9 expression and is completely reversible. Therefore, our system will serve as a useful research model to study Ub pool dynamics.

Our system can also be expanded to study the stress resistance of cells. Using our system, we can identify the types of cellular stress that can be ameliorated by prior upregulation of *UBC*. Excess Ub may play a protective role in such stress conditions, and may help maintain cellular integrity, even under stress conditions. Alternatively, excess Ub under normal conditions may not be related to the stress response. Under stress conditions, it may be possible to further upregulate *UBC* that has already been upregulated due to cellular stress, although it is unclear whether this further upregulation is directly related to an increase in the stress response. However, based on our data, it is challenging to increase *UBC* expression under normal conditions, as its basal expression levels are already high. Therefore, further upregulation of *UBC* expression under stress conditions may be even more challenging, as its expression levels are then higher.

Epigenomic states and chromatin structures are known to be essential regulators of gene expression and are dynamically regulated in response to diverse environmental stimuli [29,30]. In general, histone H3 is most frequently modified and the types of H3 modification enable us to predict whether the chromatin structure is open (euchromatin) or closed (heterochromatin). Depending on the type of H3 modification, diverse functional elements of the genome, such as promoters, enhancers, and gene bodies, can be distinguished and it can be determined whether these elements are in an active or inactive state [31]. Since the ENCODE (Encyclopedia of DNA Elements) database was launched in 2003, it has been updated with a large volume of data regarding the epigenome in diverse cell lines, tissues, and organisms [32,33]. Therefore, for effective upregulation, it is now more practical to design sgRNAs based on the epigenomic state of the gene. Using the ENCODE database, we confirmed that the sgRNAs used in this study were targeted to an intron region with an open conformation and therefore, the sgRNA binding sites were readily accessible. For effective upregulation of target genes, sgRNA binding sites should be located in a region with an open conformation, so that the bulky dCas9-sgRNA complex does not have accessibility issues.

Although transcriptional regulation of *UBC* has been widely studied, the exact promoter region of *UBC* has been difficult to define [34]. It is possible that *UBC* does not have a well-defined promoter, but has a broad promoter region [35]. Furthermore, when we analyzed the RAMPAGE (RNA Annotation and Mapping of Promoters for Analysis of Gene Expression) datasets in the ENCODE database, we found that there are 14 splice variants of *UBC* [32,33]. Among these splice variants, the start site of the non-coding exon, that is, the transcriptional start site (TSS), is variable (see Figure 1C). Based on the NCBI Reference Sequence NM_021009.6, the size of the non-coding exon is 455 bp, while based on NM_021009.7, it is 64 bp. These two major splice variants have the same intron region of 812 bp (see Figure 1C). However, many minor splice variants have their non-coding exons located further downstream, which results in a smaller intron. Interestingly, many splice variants do not contain the full sequence of the coding region and therefore, it is currently unclear whether these splice variants make any significant contributions towards the Ub pool. To accommodate all of these variants, we designed sgRNAs targeting a region upstream of the coding exon, rather than the highly variable region upstream of the non-coding exon. Therefore, sgRNAs were targeted to the intron region and specifically, to the intron region close to the coding exon (−16, −136, −243 and −157 relative to the ATG site, which is located 3 bp downstream of the coding exon start site). The presence of transcriptional activator Sp1/Sp3 binding sites in this intron region has also been reported previously [36], which supports our strategy of targeting sgRNA to this region.

Since the CRISPR activation (CRISPRa) system was first introduced in mammals, many genes have been successfully targeted for their upregulation [37]. In those studies, most target genes had low basal expression levels. The CRISPRa system can also be used to upregulate genes that are not otherwise fully transcribed. Therefore, the upregulation of genes that are already highly expressed is challenging and this has not been reported extensively, most likely due to the limitations of the system [38,39]. Although polyubiquitin gene expression levels differ spatially and temporally, their expression levels are generally high [7,19]. In this study, we searched for sgRNAs that can upregulate the polyubiquitin gene *UBC* and one sgRNA targeting the intron (sg*UBC* #3) was found to upregulate *UBC* more than 2-fold. To achieve this upregulation, we used a VP64 activator fused to dCas9, as well as p65 and HSF1 activators fused to the MS2 protein, which recognizes MS2 aptamer loops in sgRNAs [40,41]. In our transient expression system, Dox induction for 2 days was necessary and sufficient to increase the levels of dCas9, but further induction did not result in continuous dCas9 expression.

## 4. Materials and Methods

### 4.1. Cell Culture

HEK293T cells (#632180; modified for optimal production of lentivirus; Clontech, Mountain View, CA, USA) were cultured in Dulbecco’s modified Eagle medium supplemented with 10% fetal bovine serum, 20 mM glutamine, and 1% antibiotics/antimycotics at 37 °C, with 95% air and 5% CO_2_.

### 4.2. Generation of idCas9–VP64 and sgRNA Constructs

Using an online guide sequence design tool [42], we designed guide pairs located near the *UBC* promoter region, with minimal off-targeting effects, and cloned them into the *Bsm*BI site of lentiGuide-Puro (#52963; Addgene, Watertown, MA, USA) and lenti sgRNA(MS2)_puro optimized backbone (#73797, Addgene) vectors in accordance with the Zhang lab’s protocol [43,44]. All sgRNA sequences used in this study are listed in Table 1. Using the Dox-inducible Cas9 expression vector (pCW-Cas9-Puro, #50661, Addgene) and the Cas9 expression vector (lentiCas9-Blast, #52962, Addgene), we generated an inducible Cas9 expression vector with a drug resistance gene switched (pCW-Cas9-Blast) through Gibson assembly. Using the catalytically inactive Cas9 (dCas9)-VP64 expression vector (pMSCV-LTR-dCas9–VP64, #46912, Addgene) and pCW-Cas9-Blast, we generated a Dox-inducible dCas9–VP64 expression vector (pCW-dCas9–VP64-Blast) through Gibson assembly. For MS2 binding, the lenti MS2-P65-HSF1_Hygro vector (#61426, Addgene) was used.

### 4.3. Transient Transfection and Dox Treatment

HEK293T cells were seeded on poly-D-lysine-coated 24-, 12-, or 6-well plates (1 × 10^5^, 2 × 10^5^, or 5 × 10^5^ cells/well, respectively) 1 day before transfection with 1.5 μg, 3 μg, or 7.5 μg of DNA (prepared using a NucleoBond Xtra plasmid midiprep kit; #740410; MACHEREY-NAGEL, Düren, Germany), respectively, using the calcium phosphate precipitation method. At 16–18 h post-transfection, cells were washed twice with 1 × PBS and treated with the indicated concentrations of Dox (#D9891; Sigma Aldrich, St. Louis, MO, USA) or DMSO for 1–4 days.

### 4.4. Conventional and Quantitative RT-PCR

Total RNA was isolated from cultured and transfected HEK293T cells using TRI Reagent (Molecular Research Center, Cincinnati, OH, USA) following the manufacturer’s protocol. Before reverse transcription, RNA samples were treated with DNase I (amplification grade; Invitrogen, Carlsbad, CA, USA) for 15 min at room temperature. Conventional and quantitative RT-PCR (qRT-PCR) were performed as previously described [45]. Briefly, RNA concentration was measured, and 1 μg of total RNA was used as a template for reverse transcription using SuperiorScript II Reverse Transcriptase (#RT005; Enzynomics, Daejeon, Korea), and 1/20 of the resulting cDNA was used as template for conventional and qRT-PCR. To detect sgRNA expression by conventional RT-PCR, reverse transcription was performed with oligo(dT)_18_ (50 μM) and scaffold-reverse primers (5′-GAC TCG GTG CCA CTT TTT CA-3′, 20 pmoL), using Titanium Taq DNA polymerase (#639209, Clontech), with the appropriate primer pairs used for subsequent PCR. PCR-amplified samples in 1× DNA loading buffer were electrophoresed on 2% agarose gels and the images were captured using a gel documentation system (Bio-Rad, Hercules, CA, USA). The TOPreal™ qPCR 2 × PreMIX (#RT500, Enzynomics) and an iCycler system (Bio-Rad) were used for qRT-PCR. All qRT-PCR data are presented after normalization to *GAPDH* mRNA levels. The following primers were used in this study: *dCas9*-F (5′-GAG ACA AAC CAA TTC GGG AGC-3′), *dCas9*-R (5′-TCC AGC ACT TCT TTG GTG GAG-3′), *UBC*-F (5′-CCT GGT GCT CCG TCT TAG AG-3′), *UBC*-R (5′-TTT CCC AGC AAA GAT CAA CC-3′), *UBB*-F (5′-TGA GGG GTG GCT GTT AAT TC-3′), *UBB*-R (5′-CAT TTT GAA CAG GTT CAG CT-3′), *GAPDH*-F (5′-GGC CTC CAA GGA GTA AGA CC-3′), and *GAPDH*-R (5′-AGG GGT CTA CAT GGC AAC TG -3′).

### 4.5. Immunoblot Analysis

Cells were lysed in RIPA buffer and immunoblot analysis was performed as previously described [45]. Briefly, total cell lysates (15 μg) were subjected to SDS-PAGE, followed by immunoblot detection with the appropriate antibodies. Anti-Cas9 (1:500; #517386; Santa Cruz Biotechnology, Dallas, TX, USA), anti-Ub(P4D1) (1:500, #8017, Santa Cruz Biotechnology), or anti-α-Tubulin antibodies (1:500, #32293, Santa Cruz Biotechnology) were used as primary antibodies and HRP-conjugated goat anti-mouse IgG (1:10,000; #ADI-SAB-100-J; Enzo Life Sciences, Farmingdale, NY, USA) was used as the secondary antibody.

### 4.6. Statistical Analysis

A two-tailed unpaired Student’s *t*-test was used to compare data between experimental and control groups. *p* < 0.05 was considered to be statistically significant.

## Figures and Tables

**Table 1:** sgRNAs used in this study.

Target Gene	Protospacer Sequence (5′–3′)	Position Relative to 1st ATG (+1)	Reference
*UBC*	CAA AAA CGG CCA GAA TTT AG	−16	This study
	GGT TTT GAA CTA TGC GCT CG	−136	This study
	TCT CCT GAA TCG ACA GGC GC	−243	This study
	TTC TTA AGT AGC TGA AGC TC	−157	This study
*Scrambled* (Control)	GCA CTA CCA GAG CTA ACT CA	X	This study

## Supplementary Materials

Supplementary materials can be found at https://www.mdpi.com/1422-0067/20/13/3168/s1.

## Abbreviations

Ub	ubiquitin
TSS	transcriptional start site
Dox	doxycycline
HEK	human embryonic kidney
