# Supplementary material for: Reversible Regulation of Polyubiquitin Gene UBC via Modified Inducible CRISPR/Cas9 System
Source: Int J Mol Sci. 2019 Jun 28;20(13):3168. doi: 10.3390/ijms20133168 (PMC6651705; doi:10.3390/ijms20133168)
Supplement: Supplementary file 1 [file ijms-20-03168-s001.pdf]

Supplementary Figures

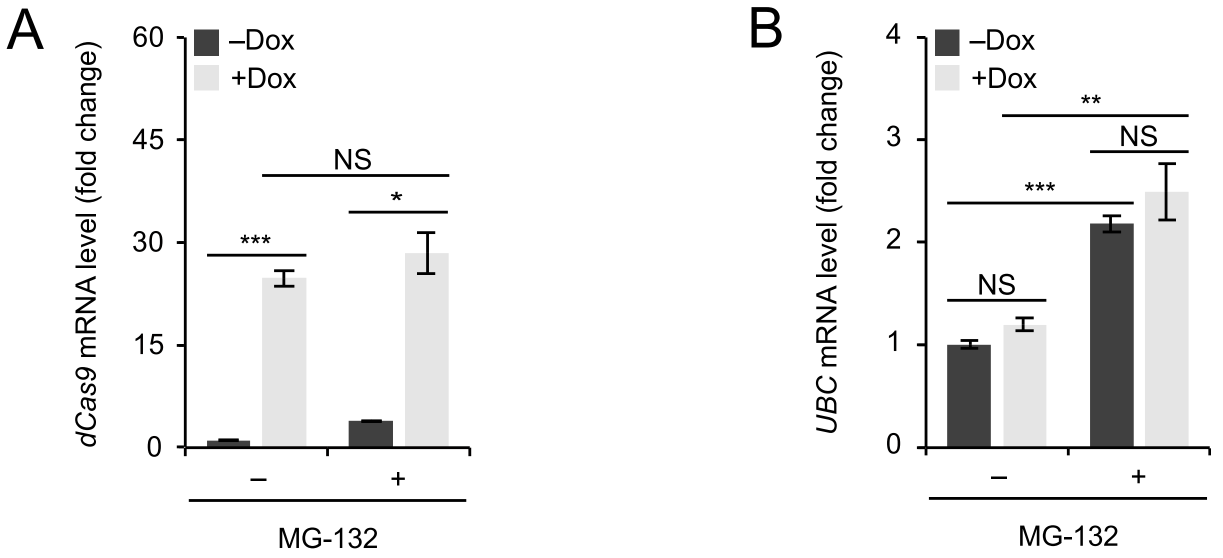

Figure S1. Validation of inducible dCas9-VP64 system under proteasome inhibition.

(A, B) HEK293T cells were transiently transfected with idCas9-VP64 and then treated with 5 μg/mL doxycycline (Dox) for 1 day. Before harvest, cells were treated with 10 μM MG-132 for 8 hr. *dCas9* and *UBC* mRNA levels were determined by qRT-PCR (n = 3 each), normalized to *GAPDH*, and expressed as the fold change relative to the control (-Dox, -MG-132). All data are presented as means ± SEM from the indicated number of samples. *$P < 0.05$; **$P < 0.01$; ***$P < 0.001$ between two groups as indicated by horizontal bars. NS: not significant.

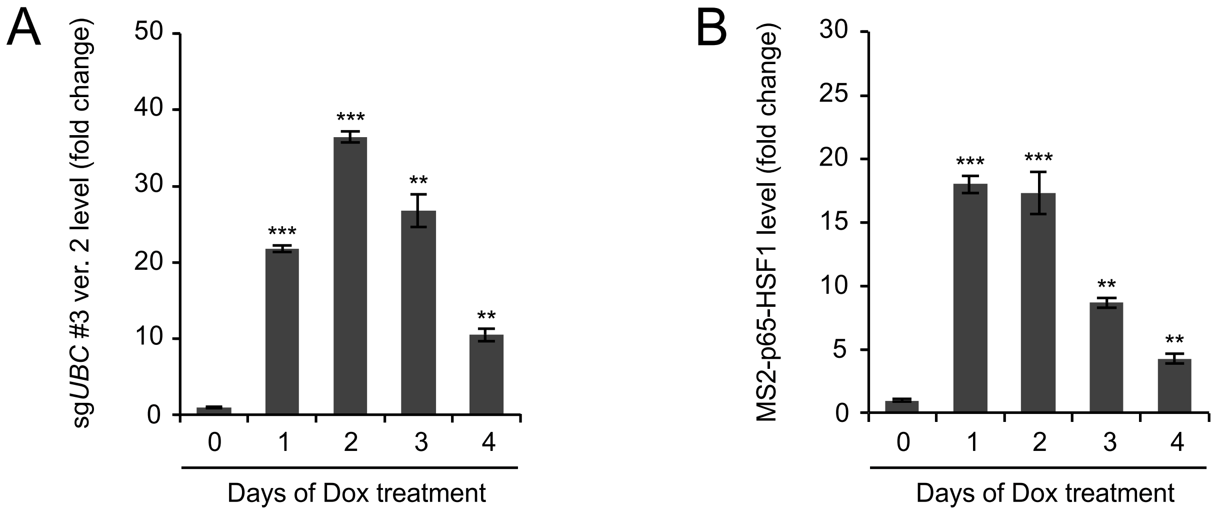

Figure S2. Time course analysis of sgRNA ver. 2 and MS2-p65-HSF1 expression in the idCas9-VP64 system.

(A, B) HEK293T cells were transiently transfected with idCas9-VP64, MS2-p65-HSF1, and sg*UBC* #3 ver. 2, and then treated with 10 µg/mL Dox for up to 4 days. sg*UBC* #3 ver. 2 and MS2-p65-HSF1 mRNA levels were determined by qRT-PCR (n = 3 each), normalized to *GAPDH*, and expressed as the fold change relative to the control (0 days of Dox treatment). To detect sg*UBC* #3, the appropriate forward primer as shown in Table 1 and the MS2 stem-loop reverse primer (5’-GGT GAT CCT CAT GTT GGC CAA G-3’) were used. The following primers were used to detect MS2-p65-HSF1 mRNA expression: MS2-p65-HSF1-F (5’-AAA AAG CCT GAA CTC ACC GC-3’) and MS2-p65-HSF1-R (5’-CAT CGA AGC TGA AAG CCC GA-3’). All data are presented as means ± SEM from the indicated number of samples. **P < 0.01; ***P < 0.001 vs. control (0 days of Dox treatment).
